# Supplementary material for: Synergistic Antioxidant Effect of Prebiotic Ginseng Berries Extract and Probiotic Strains on Healthy and Tumoral Colorectal Cell Lines
Source: Int J Mol Sci. 2022 Dec 26;24(1):373. doi: 10.3390/ijms24010373 (PMC9820163; doi:10.3390/ijms24010373)
Supplement: Supplementary file 1 [file ijms-24-00373-s001.zip › ijms-2074768-supplementary.pdf]

Table S1. Growth of strains used in this study.

Strain	mMRS (CTR)	*P. ginseng* Extract 2%	*p*-Value
B. cellulosilyticus	0.54 ± 0.07	3.45 ± 0.03	**
C. orbiscindens (formerly *F. plautii*)	0.91 ± 0.06	3.79 ± 0.05	**
C. symbiosum	0.58 ± 0.09	3.74 ± 0.02	**
E. coli	0.97 ± 0.003	3.65 ± 0.19	**
R. gnavus	0.93 ± 0.02	3.96 ± 0.14	**
B. finegoldii	0.66 ± 0.06	3.98 ± 0.36	****
L. plantarum	0.63 ± 0.05	4.75 ± 0.19	****
L. acidophilus	0.77 ± 0.19	4.40 ± 0.18	***
L. reuteri	0.85 ± 0.10	4.78 ± 0.05	**
L. fermentum	0.75 ± 0.29	5.72 ± 0.67	****
L. rhamnosus	0.78 ± 0.08	4.53 ± 0.31	**
B. longum subsp. *infantis*	0.73 ± 0.11	6.40 ± 0.20	***
B. animalis subsp. *lactis*	0.64 ± 0.23	4.48 ± 0.08	***
B. longum	0.59 ± 0.15	4.30 ± 0.04	***

Table S2. Mean values of the ratio GSH/GSSH ± Standard Error (SE) in HT-29 and CCD841 cell lines after the treatment with total Ginseng berries extract (ET), and fermented Ginseng berries extract from the probiotic consortium (EF); CTR is the control condition (i.e., not treated). The significant variations with respect to the CTR are expressed as *p*-values calculated by Dunnett's tests. ** *p*-value <0.01, * < 0.05, NS is for Not Significant.

Sample	Ratio GSH/GSSG ± SE	*p*-Value
	**HT-29**	
CTR	0.87 ± 0.04	-
ET	0.716 ± 0.001	*
EF	0.722 ± 0.001	*
	**CCD841**	
CTR	0.928 ± 0.005	-
ET	0.975 ± 0.114	NS
EF	0.756 ± 0.006	*
